# A cross-sectional study measuring contact patterns using diaries in an urban and a rural community in South Africa, 2018

**DOI:** 10.1186/s12889-021-11136-6

**Published:** 2021-06-03

**Authors:** Jackie Kleynhans, Stefano Tempia, Meredith L. McMorrow, Anne von Gottberg, Neil A. Martinson, Kathleen Kahn, Jocelyn Moyes, Thulisa Mkhencele, Limakatso Lebina, F. Xavier Gómez-Olivé, Floidy Wafawanaka, Azwifarwi Mathunjwa, Cheryl Cohen, the PHIRST group, Amelia Buys, Angela Mathee, Brigitte Language, Lorens Maake, Florette Treurnicht, Katlego Mothlaoleng, Maimuna Carrim, Nicole Wolter, Orienka Hellferscee, Ryan G. Wagner, Stuart Piketh

**Affiliations:** 1 Centre for Respiratory Diseases and Meningitis, National Institute for Communicable Diseases of the National Health Laboratory Service, Johannesburg, South Africa; 2 School of Public Health, Faculty of Health Sciences, University of the Witwatersrand, Johannesburg, South Africa; 3 Influenza Division, Centers for Disease Control and Prevention, Atlanta, GA USA; 4 Influenza Program, Centers for Disease Control and Prevention, Pretoria, South Africa; 5 MassGenics, Duluth, Georgia USA; 6 United States Public Health Service, Rockville, MD USA; 7 School of Pathology, Faculty of Health Sciences, University of the Witwatersrand, Johannesburg, South Africa; 8 Perinatal HIV Research Unit (PHRU), University of the Witwatersrand, Johannesburg, South Africa; 9 Johns Hopkins University Center for Tuberculosis Research, Baltimore, MD USA; 10 Department of Science and Technology/National Research Foundation Centre of Excellence for Biomedical Tuberculosis Research, University of the Witwatersrand, Johannesburg, South Africa; 11 MRC/Wits Rural Public Health and Health Transitions Research Unit (Agincourt), School of Public Health, Faculty of Health Sciences, University of the Witwatersrand, Johannesburg, South Africa; 12 Environment and Health Research Unit, South African Medical Research Council, Johannesburg, South Africa; 13 Unit for Environmental Science and Management, School of Geo- and Spatial Science, North-West University, Potchefstroom, South Africa

**Keywords:** Contact diaries, Urban, Rural, Infectious disease modelling

## Abstract

**Background:**

Describing contact patterns is crucial to understanding infectious disease transmission dynamics and guiding targeted transmission mitigation interventions. Data on contact patterns in Africa, especially South Africa, are limited. We measured and compared contact patterns in a rural and urban community, South Africa. We assessed participant and contact characteristics associated with differences in contact rates.

**Methods:**

We conducted a cross-sectional study nested in a prospective household cohort study. We interviewed participants to collect information on persons in contact with for one day. We described self-reported contact rates as median number people contacted per day, assessed differences in contact rates based on participant characteristics using quantile regression, and used a Poisson model to assess differences in contact rates based on contact characteristics within age groups. We also calculated cumulative person hours in contact within age groups at different locations.

**Results:**

We conducted 535 interviews (269 rural, 266 urban), with 17,252 contacts reported. The overall contact rate was 14 (interquartile range (IQR) 9–33) contacts per day. Those ≤18 years had higher contact rates at the rural site (coefficient 17, 95% confidence interval (95%CI) 10–23) compared to the urban site, for those aged 14–18 years (13, 95%CI 3–23) compared to < 7 years. No differences were observed for adults. There was a strong age-based mixing, with age groups interacting more with similar age groups, but also interaction of participants of all ages with adults. Children aged 14–18 years had the highest cumulative person hours in contact (116.3 rural and 76.4 urban).

**Conclusions:**

Age played an important role in the number and duration of contact events, with children at the rural site having almost double the contact rate compared to the urban site. These contact rates can be utilized in mathematical models to assess transmission dynamics of infectious diseases in similar communities.

**Supplementary Information:**

The online version contains supplementary material available at 10.1186/s12889-021-11136-6.

## Introduction

Contact patterns within communities impact disease transmission and may inform development of interventions to reduce transmission [1]. To evaluate the impact of both pharmaceutical and non-pharmaceutical interventions on disease transmission, mathematical models are utilized when intervention studies may be unethical or too expensive to perform [2]. Mathematical modelling results are only as reliable as the data used for parametrization and the validity of the assumptions made. Disease transmission models usually require accurate age-related contact patterns [3].

Although sub-Saharan Africa has the highest burden of infectious diseases globally, [4] data on contact patterns within the region are limited, making modelling infectious disease transmission challenging [5]. Contact studies have been carried out in Kenya, [6, 7] Uganda, [8, 9] Senegal, [10] Zimbabwe [11] and South Africa [12, 13]. The South African studies were performed in Cape Town communities, and it is unknown how contact patterns reported in Cape Town compare to other South African communities.

The Prospective Household cohort study of Influenza, Respiratory Syncytial virus and other respiratory pathogens community burden and Transmission dynamics in South Africa (PHIRST study) was a three-year, randomly selected, household-level community cohort study conducted at two sites, from 2016 through 2018 [14]. The primary objectives of the study were to estimate the community burden of and assess the transmission dynamics of influenza, respiratory syncytial virus (RSV), *Bordetella pertussis* and *Streptococcus pneumoniae* colonization. To better understand the role of contact patterns on the transmission of these organisms, we nested a contact diary study in the final year of the PHIRST study.

We aimed to measure and compare contact patterns in a rural and an urban community in South Africa using interviewer completed contact diaries, and to assess participant and contact characteristics that influence differences in contact rates.

## Methods

### Study population

From August to October 2018, participants in a prospective household cohort study in two sites were invited to participate in the contact survey. One hundred and seventeen households, 56 households with 282 participants at the rural site, and 61 households with 285 participants at the urban site, were included. In short, PHIRST was a three-year household-level community cohort study conducted in a rural community in Bushbuckridge Municipality (Mpumalanga Province) and an urban area in the Matlosana Municipality (North West Province) from 2016 through 2018. A random selection of approximately 50 households were enrolled each year from 2016 through 2018. The rural site formed part of a health and socio-demographic surveillance site (HDSS) at the Medical Research Council (MRC)/University of Witwatersrand Rural Public Health and Health Transitions Research Unit, Agincourt. At the rural site, a list of households with 3 or more members were requested from the HDSS and at the urban site, a list of 450 global positioning system (GPS) coordinates within the Jouberton geographical area was generated. Households from these lists were approached sequentially and were eligible for enrolment if it consisted of 3 or more household members (sharing at least 4 meals a week) and > 80% of household members consented to participant in the study. As part of the main study, households were visited twice weekly for 8–10 months for collection of upper respiratory specimens, symptom and healthcare consultation information from all participating household members. Sample size calculations for the cohort study were based on the primary objective that focussed on transmission dynamics of influenza and RSV, and was based on 10% risk of infection, with 95% confidence intervals and 5% precision [14].

### Data collection

We interviewed participants of the PHIRST study once to ascertain information on all the persons they were in close contact with for one day. For each participant’s contact diary, we collected age (or age group if age in years was unknown: < 7, 7–13, 14–18, 19–64, or ≥ 65 years); sex; if contact occurred in enclosed space or not; and whether there was physical contact for each person contacted on the day. We also ascertained if the contact was a member of the participant’s household; total time spent with the contact during the day (< 5 min, 5–14 min, 15–59 min, 1–4 h, > 4 h); where the contact took place (home, school, work, transport or other) and how often the participant came into contact with the person (daily/almost daily, once or twice a week, once or twice a month, less than once a month, or never met before).

We assigned each household a random day of the week to measure contact events. Fieldworkers visited each household the day prior to the measurement day to explain study procedures to all household members. Fieldworkers informed participants of what information would be collected, provided a blank notebook and pen and encouraged participants to make notes that might be of use during the interview. The day following the measurement day, the field worker visited the household again and conducted an interview with each participant to complete the contact survey (Additional file 4). A time use survey was completed at the same time to assist with recall of contacts. If the entire household or a participant within the household was not available for interview on the selected day, a more convenient day was chosen, based on the age distribution of individuals allocated to the days of the week, giving preference to ensure that there were children aged < 5 years and elderly individuals ≥65 years allocated to each day of the week. The investigation spanned from 14 August – 14 October and 2 August – 26 September at the rural and urban site, respectively. There were 2 public holidays within this period, which were not investigated for any participants. The school holiday was from 29 September – 8 October, in which period no investigations were done, except for 9 surveys from the rural site performed on the first day of the holiday which fell on a Saturday. We collected both contact events within the household and the community using the contact diaries and captured data on a REDCap database [15, 16].

### Contact classification and contact rates

We investigated contact rate as the primary outcome, which we defined as the median number of people contacted per day per group (based on characteristics of the study participant such as age, sex, etc.). A contact was defined as a two-way conversations of at least three words at a distance not requiring voices being raised (< 2 m between individuals), with or without physical contact, or where physical contact took place without conversation. We further classified contacts as physical and non-physical, where physical contacts included kissing, touching, or hand shaking.

We analyzed the data using Stata 14.2 (StataCorp, College Station, Texas, USA). We accounted for within-household clustering using the Taylor-linearised variance estimation (“svy” Stata function) when calculating contact rates. Ninety-five percent confidence intervals for median contact rates were obtained using the ‘epctile’ function. Stratified contact rates are only presented where data were available for four or more participants within the stratum.

### Participant characteristics associated with contact rate

To assess differences in contact rates based on participant characteristics, or day investigated, we used quantile regression, with 500 bootstrap replications and accounting for clustering at site and household level [17]. Quantile regression allows univariate and multivariable comparison of percentiles (including medians) between groups or categories [18]. This analytical approach was selected a priori due to the right skewed nature of contact data. Contact events are not normally distributed, but rather follow a power-low distribution where several individuals have few contacts and a few have large numbers of contacts [19], making the use of linear regression unsuitable for the analysis of contact events. The quantile regression model outputs a coefficient that quantifies the increase/decrease in median number of contact events for each category of the evaluated predictor when compared to the reference category. Predictors were first assessed on univariate analysis, and those with a *p*-value of < 0.2 were included in multivariable analyses. We did separate analysis for children (aged 0–18 years) and adults (≥19 years). We assessed site, age, relationship to head of household, sex, if day investigated was a weekday (Monday-Friday) or weekend (Saturday/Sunday), day of the week investigated and HIV status. For adults we also assessed education, employment, alcohol consumption, and cigarette smoking. Backwards elimination was done by excluding categories with the highest *p*-values until all remaining predictors had a p-value of < 0.05.

### Age-related mixing patterns and contact characteristics associated with differences in contact rates

To investigate age-related mixing patterns, we calculated the total number of contacts each age group had with a specified age group (< 7, 7–13, 14–18, 19–64 and ≥ 65 years) and then calculated the proportion of contacts with that age group out of total contacts for the day.

To assess differences in contact rates based on the characteristics of the contact (contact type, contact with a household member, duration of contact, frequency of contact and location of contact), we utilized a Poisson model for each age group described above. Lack of overdispersion was assessed using a negative binomial model, hence a Poisson model was retained for the analysis.

### Cumulative person hours in contact

As a secondary outcome, we estimated the total time spent in contact with persons. We assigned a minute value to each category of total time spent with the contact, based on the mid-point for the first 4 categories, and the lower point for the last. We assigned: < 5 min = 2.5, 5–14 min = 7.5, 15–59 min = 30, 1–4 h = 120, > 4 h = 240. For each age category and location, the contact took place we calculated cumulative person hours in contact = (summed total minutes for each contact / participant in age category)/60. Since contact could have been with multiple people simultaneously, the time spent in contact could be more than the 24-h reporting period. Where more than one location was selected for the contact, the time spent counted towards both locations.

## Results

### Study population

We conducted 535 interviews from August through October 2018, representing 95% of the 2018 PHIRST cohort (*N* = 565), of which 269/280 (96%) were conducted in the rural, and 266/285 (93%) in the urban site (Additional file 1). The average household size was five individuals at each site (range 3–11 at the rural site; 4–10 at the urban site). There were more females (*n* = 337, 63%) included than males. Most adults were unemployed (127/225, 56% Additional file 2). The most common relationship to the head of the household was being their child (165/423, 39%), followed by being a grandchild (91/423, 22%).

### Contact rates

**Fig. 1:**
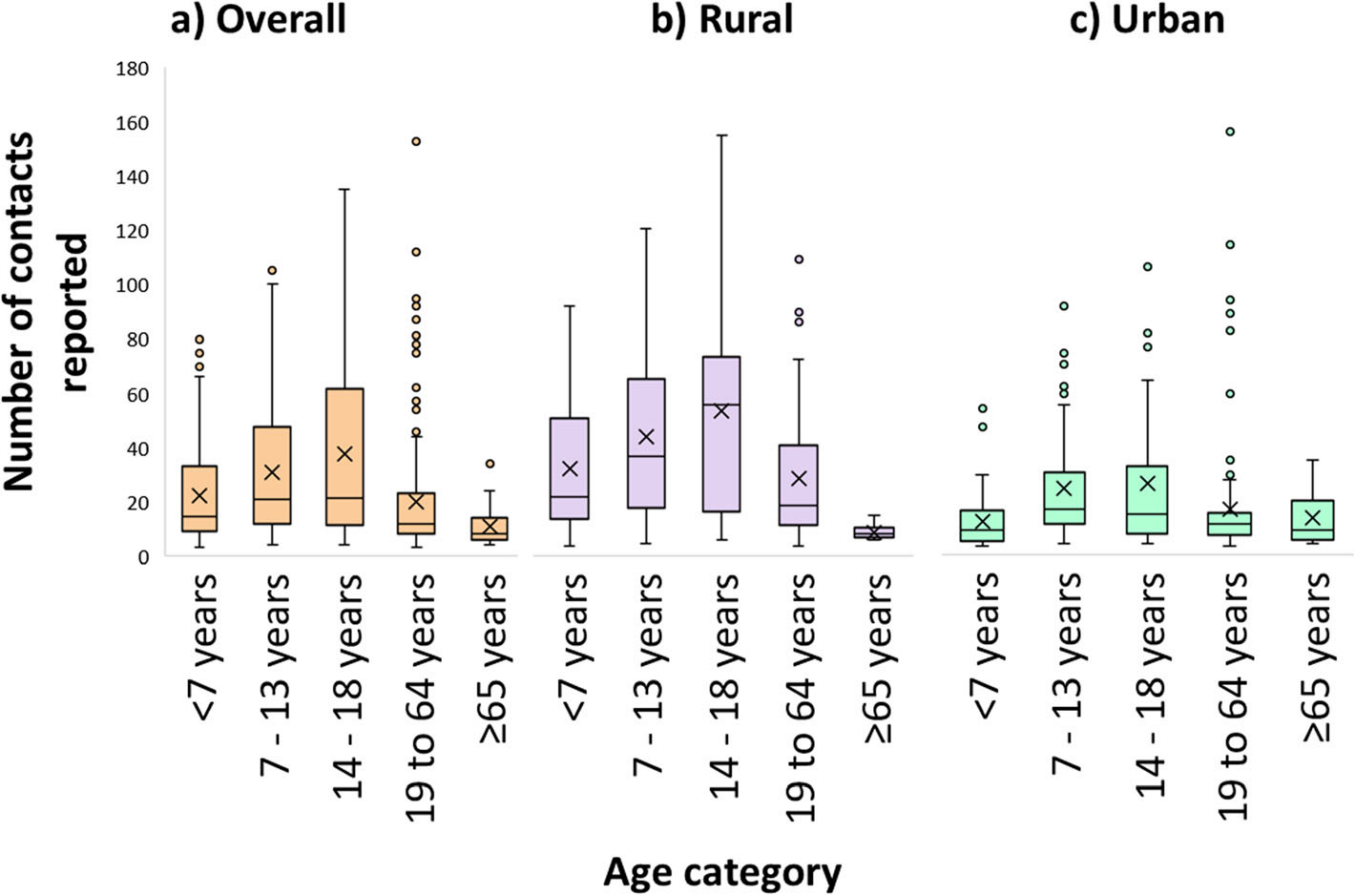
Contact rate by age group, South Africa, 2018. Physical and conversational contacts (**a**) overall, (**b**) rural and (**c**) urban site. Line represents the median, cross represents the average, box represents the 25th and 75th percentile, whiskers represent 1st and 99th percentile, circles indicate outliers

Overall, 17,252 contacts were reported by 535 participants, of which 59% (10,304) were reported from the rural site. Many individuals reported a low number of contacts and few individuals reported a high number of contacts. The overall contact rate was 14 (IQR 9–33, Fig. 1, Additional file 2), with the highest number of contacts from one participant reported as 153. There were 58/269 (21.6%) participants at the rural site and 20/266 (7.5%) at the urban site that reported more than 50 contacts for the day. We observed a higher contact rate at the rural site compared to the urban site (21, IQR 11–46 vs 12, IQR 7–18, *p* = 0.001). The highest contact rate was observed in children aged 14–18 years, with a contact rate of 22 contacts (IQR 12–91) per day (Fig. 1, Additional file 2). For both sites, higher contact rates were reported for Wednesdays and Thursdays (Additional file 2).

### Participant characteristics associated with contact rate

Using quantile regression (median difference) site, age and day of the week investigated were found to be associated with differences in contact rates for children (aged ≤18 years, Table 1). There was a higher contact rate at the rural site compared to the urban site (coefficient 17, 95% CI 10 to 24). Compared to those < 7 years old, children 7–13 years and 14–18 years had a higher contact rate (9, 95% CI 4–14 and 13, 95% CI 4–22). Compared to Saturdays, there were more contacts on Wednesdays (18, 95% CI 4–32) and Thursdays (12, 95% CI 1–23). HIV status was not associated with number of contacts for children or adults. For adults there were no factors associated with number of contacts on multivariable analysis (Table 2).

**Table 1:** Participant characteristics associated with differences in median number of people contacted for participants ≤18 years, South Africa, 2018

	Univariate		Multivariable	
	Coefficient	95% CI	Coefficient	95% CI
**Site**				
Rural	15	3 to 27	17	10 to 24
Urban	Reference	–	Reference	–
**Age**				
< 7 years	Reference	–	Reference	–
7–13 years	6	1 to 11	9	4 to 14
14–18 years	7	− 7 to 21	13	4 to 22
**Relationship to head of household**				
Grandchild	1	− 11 to 13		
Child	2	− 10 to 14		
Other child*	Reference	–		
**Gender**				
Male	2	−4 to 8		
Female	Reference	–		
**Type of day investigated**				
Weekend day	Reference	–		
Weekday	12	3 to 21		
**Day of week investigated**				
Monday	5	−1 to 11	1	−7 to 9
Tuesday	7	−4 to 18	2	−7 to 11
Wednesday	35	7 to 63	18	4 to 32
Thursday	27	11 to 43	12	1 to 23
Friday	9	−6 to 24	Omitted**	–
Saturday	Reference	–	Reference	–
Sunday	3	0 to 6	1	−4 to 6
**HIV Status**				
Negative	Reference	–		
Positive	5	−28 to 38		

95% CI – 95% confidence interval

*Other child – Includes relationships of participants younger than 18 years to the head of household (n): brother (10), sister (11), wife (1), cousin (5), niece (6), nephew (9), other relative (10), not related (2)

**omitted because of collinearity

**Table 2:** Participant characteristics associated with differences in median number of people contacted for participants > 18 years, South Africa, 2018

	Univariate	
	Coefficient	95% CI
**Site**		
Rural	2	0 to 4
Urban	Reference	–
**Age**		
19–64 years	2	0 to 4
≥65 years	Reference	–
**Relationship to head of household**		
Head of home	3	−1 to 7
Parent	Reference	–
Grandchild	3	−3 to 9
Child	3	−1 to 7
Other adult*	2	−2 to 6
**Gender**		
Male	0	−2 to 2
Female	Reference	–
**Type of day investigated**		
Weekend day	2	0 to 4
Weekday	Reference	–
**Day of week investigated**		
Monday	1	−3 to 5
Tuesday	1	−2 to 4
Wednesday	3	−1 to 7
Thursday	2	−2 to 6
Friday	Reference	–
Saturday	4	0 to 8
Sunday	3	−1 to 7
**Education**		
No schooling	2	0 to 4
Primary	1	−1 to 3
Some secondary	Reference	–
Completed secondary	2	0 to 4
Post-secondary	3	−1 to 7
**Employment**		
Employed	2	0 to 4
Unemployed	Reference	–
**Drinking alcohol**		
No	2	1 to 3
Yes	Reference	–
**Smoking cigarettes**		
No	2	1 to 3
Yes	Reference	–
**HIV Status**		
Negative	1	0 to 2
Positive	Reference	–

95% CI – 95% confidence interval

* Other adult – Includes relationships of participants 18 years and older to the head of household (n): brother (7), sister (7), wife (19), husband (4), cousin (1), niece (4), nephew (2), other relative (3), not related (4)

### Age-related mixing patterns and contact characteristics associated with differences in contact rates

**Fig. 2:**
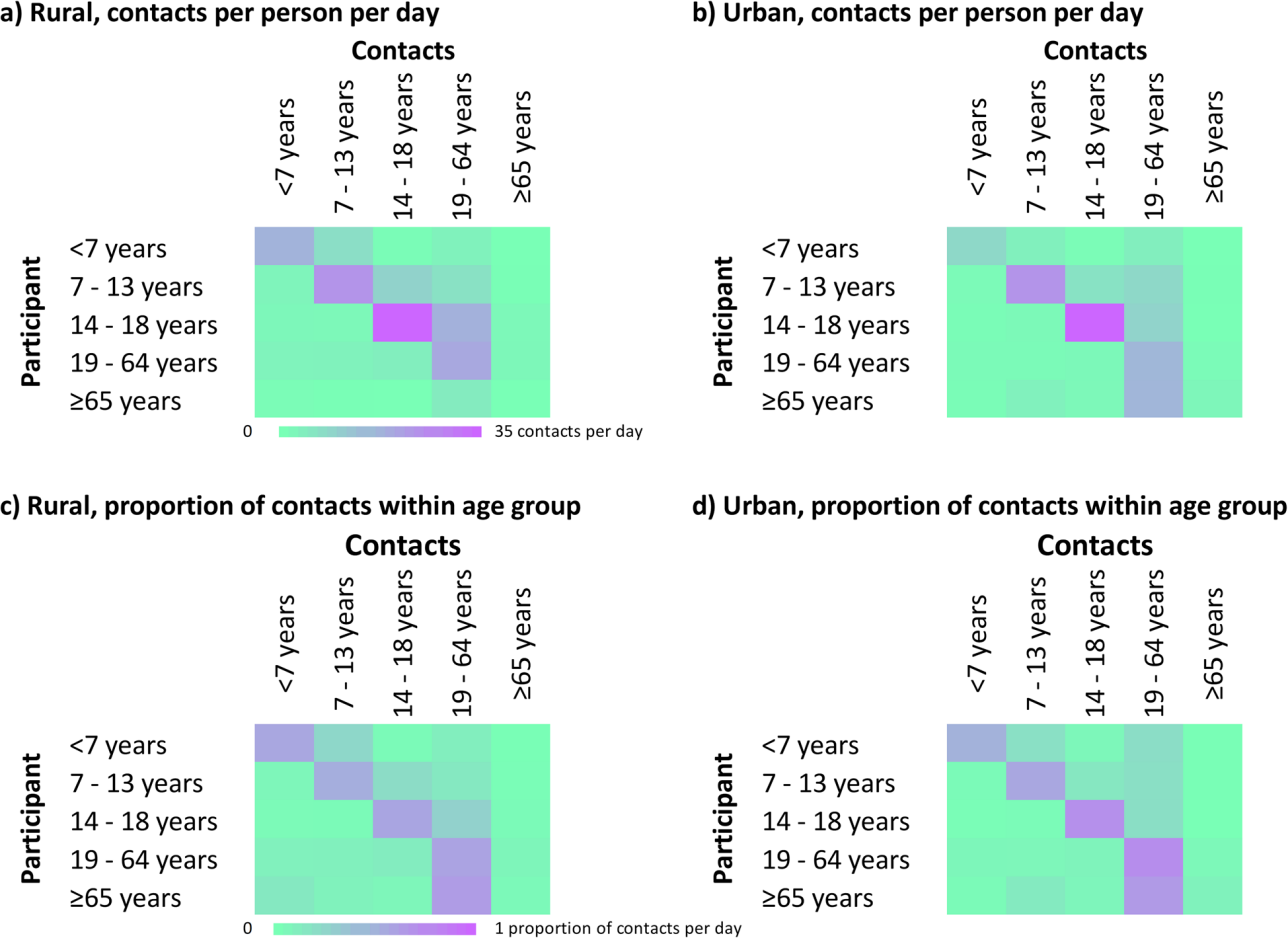
Age-related contact matrix for total number of people contacted, South Africa, 2018. Physical, conversational and confined contacts by (**a**, **c**) rural and (**b**, **d**) urban site, showing (**a**, **b**) number of contacts and (**c**, **d**) proportion of contacts within age groups, with participant age on y axis and contact age on x axis

There was a strong age-based assortment (mixing), with age groups interacting with similar age groups, but also a higher interaction of participants of all ages with other adults (Fig. 2).

Using Poisson regression, we found children aged < 7 years were more likely to have contact with children their own age (risk ratio (RR) 8.2, 95%CI 5.8–11.6) than those aged ≥65 years, to have contact with the same people daily (RR 16.1 95%CI 6.7–38.7) compared to once or twice a month, and to have longer contacts (> 4 h, RR 2.9 95%CI 1.9–4.4) compared to those lasting < 5 min (Table 3). Children aged 7–13 and 14–18 years had a similar contact profile, except for being more likely to have contact with household members (RR 1.3 95%CI 1.2–1.4 and RR 1.2 95%CI 1.1–1.4, respectively) than non-household members, and those aged 14–18 years being less likely to have contacts of 1–4 h duration (RR 0.6 95%CI 0.5–0.8) compared to contacts lasting < 5 min. Adults aged 19–64 years also had shorter contacts and were less likely to see people daily compared to once or twice a month (RR 0.3 95%CI 0.2–0.3). Adults ≥65 years were more likely to come into contact with someone they never met before (RR 5.8 95% CI 1.8–18.9) than someone they see once or twice a month, and less likely to have contacts at home (RR 0.3 95%CI 0.2–0.4) than in public transport (Table 3).

**Table 3:** Contact characteristics associated with differences between contact rates within age groups, South Africa, 2018

	Risk ratio (95% confidence interval)					
	All ages	< 7 years	7–13 years	14–18 years	19–64 years	≥65 years
**Type of contact**						
Physical	**1.2 (1.1 to 1.2)**	**1.5 (1.4 to 1.6)**	1.0 (0.9 to 1.0)	**0.8 (0.7 to 0.9)**	0.9 (0.9 to 1.0)	**0.3 (0.2 to 0.4)**
Non-physical	Reference					
**Age of contact**						
< 7 years	**6.1 (5.5 to 6.8)**	**8.2 (5.8 to 11.6)**	**0.6 (0.4 to 0.8)**	**0.5 (0.3 to 0.8)**	**0.3 (0.2 to 0.3)**	**0.3 (0.1 to 0.7)**
7–13 years	**8.5 (7.7 to 9.5)**	**2.9 (2.0 to 4.1)**	**3.5 (2.7 to 4.4)**	**0.4 (0.2 to 0.6)**	**0.2 (0.2 to 0.2)**	0.4 (0.2 to 1.0)
14–18 years	**6.7 (6.0 to 7.5)**	**0.6 (0.4 to 0.8)**	**1.8 (1.4 to 2.4)**	**6.3 (4.4 to 9.1)**	**0.2 (0.2 to 0.3)**	**0.2 (0.1 to 0.5)**
19–64 years	**11.9 (10.7 to 13.2)**	1.2 (0.8 to 1.7)	0.9 (0.7 to 1.1)	**1.9 (1.3 to 2.8)**	0.9 (0.8 to 1.0)	1.3 (0.6 to 2.9)
≥65 years	Reference					
**Gender of contact**						
Male	Reference					
Female	1.2 (1.1 to 1.2)	1.0 (0.9 to 1.1)	0.8 (0.8 to 0.9)	1.0 (0.9 to 1.1)	**1.2 (1.1 to 1.3)**	1.1 (0.8 to 1.5)
Not reported	0.1 (0.1 to 0.1)	1.4 (1.2 to 1.7)	0.4 (0.3 to 0.5)	1.2 (0.9 to 1.5)	1.3 (1.1 to 1.6)	NEO
**Contact is household member**						
No	**3.9 (3.7 to 4.0)**	0.9 (0.8 to 1.0)	**1.3 (1.2 to 1.4)**	**1.2 (1.1 to 1.4)**	**0.8 (0.8 to 0.9)**	**0.4 (0.3 to 0.5)**
Yes	Reference					
**Total time spent with contact during the day**						
< 5 m	Reference					
5-15 m	**2.5 (2.2 to 2.9)**	1.3 (0.8 to 2.1)	1.4 (1.0 to 2.1)	0.8 (0.6 to 1.1)	0.9 (0.8 to 1.1)	0.6 (0.2 to 1.8)
15 m-1 h	**8.7 (7.7 to 9.9)**	**2.0 (1.3 to 3.1)**	**1.6 (1.1 to 2.2)**	0.8 (0.6 to 1.1)	**0.8 (0.7 to 0.9)**	0.8 (0.3 to 2.2)
1-4hts	**16.8 (14.8 to 19.1)**	**2.9 (1.9 to 4.5)**	**2.5 (1.8 to 3.5)**	**0.6 (0.5 to 0.8)**	**0.5 (0.4 to 0.6)**	0.7 (0.3 to 1.7)
> 4 h	**22.1 (19.5 to 25.0)**	**2.9 (1.9 to 4.4)**	**2.3 (1.6 to 3.2)**	1.0 (0.7 to 1.3)	**0.4 (0.4 to 0.5)**	0.5 (0.2 to 1.3)
**Frequency of contact**						
Daily/almost daily	**34.6 (30.8 to 38.8)**	**16.1 (6.7 to 38.7)**	**8.9 (4.9 to 16.1)**	1.4 (1.0 to 2.0)	**0.3 (0.2 to 0.3)**	0.8 (0.3 to 2.7)
Once or twice a week	**5.7 (5.1 to 6.5)**	**5.5 (2.2 to 13.3)**	**4.4 (2.4 to 8.1)**	1.3 (0.9 to 1.9)	**0.7 (0.6 to 0.8)**	2.4 (0.8 to 7.9)
Once or twice a month	Reference					
< 1 a month	**0.7 (0.6 to 0.9)**	0.8 (0.2 to 3.5)	0.4 (0.1 to 1.3)	No observations	1.2 (1.0 to 1.4)	0.5 (0.0 to 4.4)
Never met before	**1.8 (1.6 to 2.1)**	**3.9 (1.5 to 10.0)**	**4.5 (2.4 to 8.4)**	**0.5 (0.3 to 0.8)**	**0.8 (0.7 to 0.9)**	**5.8 (1.8 to 18.9)**
**Location of contact**						
Home	**3.6 (3.3 to 3.9)**	1.2 (1.0 to 1.5)	**2.1 (1.6 to 2.8)**	1.1 (0.8 to 1.5)	**0.8 (0.7 to 0.9)**	**0.3 (0.2 to 0.4)**
School	**8.2 (7.7 to 8.8)**	**1.5 (1.2 to 1.8)**	**3.7 (2.8 to 4.9)**	**2.6 (2.0 to 3.5)**	0.1 (0.1 to 0.1)	No observations
Work	**0.8 (0.7 to 0.8)**	No observations	0.0 (0.0 to 0.1)	0.0 (0.0 to 0.1)	2.0 (1.7 to 2.3)	0.0 (0.0 to 0.1)
Transport	Reference					
Other	**5.1 (4.7 to 5.5)**	0.8 (0.6 to 1.0)	**2.2 (1.7 to 2.9)**	1.2 (0.9 to 1.7)	0.9 (0.8 to 1.1)	0.1 (0.1 to 0.1)

Cells indicate risk ratio with 95% confidence interval in parenthesis. Reference group chosen as category with least number of contacts when combining all ages, except where lowest number were in a category where some age groups have zero contacts, then second lowest used (frequency)

### Cumulative person hours in contact

**Fig. 3:**
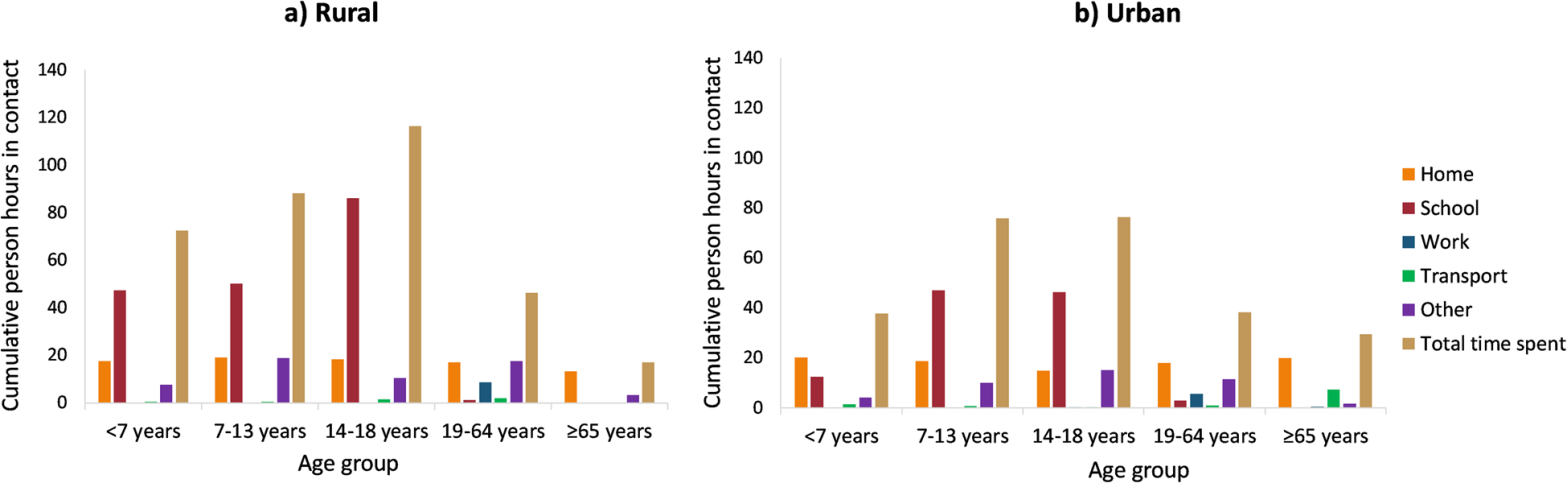
Cumulative person hours in contact reported by age, location and site, South Africa, 2018

A total of 339.8 and 257.5 cumulative person hours in contact were reported for the rural and urban site, respectively. Children aged 14–18 years at both sites had the highest cumulative person hours in contact (116.3 h rural and 76.4 h urban, Fig. 3 and Additional file 3). For children between 7 and 18 years the most person hours in contact were at school (75.9–116.3 h). Children < 7 years at the rural site spent more time at school (47.1 h) compared to home (17.4 h), whereas those at the urban site had 12.4 person hours in contact at school and 20.0 person hours in contact at home. Adults (19–64 years) had most of their person hours in contact at home (rural 17 h, urban 17.8 h) or another location (rural 13.7 h and urban 11.5 h). The elderly (≥65 years) had the lowest number of person hours in contact of all the ages, most being at home (rural 13.4 h, urban 19.8 h). The elderly at the urban site had the highest person hours in contact during transport (7.4 h) than other ages.

## Discussion

In this study including participants from randomly selected households, site, age and the day of the week were independently associated with differences in contact rates in children ≤18 years, estimated with contact diary survey data representing > 17,000 contacts. We observed higher contact rates among participants in the rural site, among children aged less than 14 years and on Wednesdays and Thursdays. Children reported seeing the same individuals daily, whereas adults and the elderly had more variation in how often they were in contact with the same individual.

The difference contact rates observed between the rural and urban site (21 and 12, respectively) in our study is in contrast to what was seen in Zimbabwe where individuals at the urban site had a greater number of contacts than the rural site (median of 11 vs. 10, respectively). Although the number of contacts at the urban site for both South Africa and Zimbabwe was similar, there was a lower number of contacts at the rural site in Zimbabwe compared to South Africa. This could also be influenced by cultural, household composition and geographical differences between the sites. We observed that these differences were driven by children. The contact rate recorded in a prior study of the Cape Town community in South Africa was more comparable to the rural site, with 20 in Cape Town compared to 21 contacts at the rural and 12 at the urban site in our study [13].

A contact study performed in rural Uganda found the mean number of contacts per person as 7, [8] and in Kenya 18 contacts were observed per day, the trend of having more contacts in a rural setting compared to a semi urban setting was also observed [6]. This highlights the variability that can be observed in contact rates between different settings, and most likely the underlying culture and social-demographic situation may dictate contact rates, but could also be attributed to differences in study methods. Surveys where participants are informed in advance of information that will be collected generally results in less recall bias, and self-completed contact diaries can allow a large sample size as less interviewers are needed, but have a lower response rate [20].

We observed a strong age-based mixing pattern as with many prior contact studies, [6, 11, 21] which was most pronounced for the 14 to 18 year old age group. The highest contact rate was observed in children, especially those of primary school age (7–13 years). This also holds true for studies done in Britain, [22] China, [21] Kenya [6] and Zimbabwe [11]. Certain age groups were also more or less likely to have contact with other age groups, have contact with individuals for certain durations and frequencies and have contacts at certain locations. This information can be useful when looking at targeted intervention strategies to reduce disease transmission in specific age groups, for example providing transport for the elderly where safe physical distancing can be practiced.

The cumulative person hours in contact was also highest in children and dominated by school contacts. Schools have previously been described as a focal point for contact and transmission events for respiratory viruses [23–25]. School closure has been shown to reduce influenza transmission – whether done so for holidays or in response to an outbreak [26–28]. However, the implementation of reactive school closures is challenging, and comes at a social and economic cost [28]. Adaption of schools to accommodate physical and social distancing is a promising alternative [29]. During the coronavirus disease 2019 (COVID-19) pandemic, school closures were also used as part of a multi-faceted approached to curb the spread of the Severe acute respiratory syndrome coronavirus 2 (SARS-CoV-2), [30, 31] although it seems that specifically for SARS-CoV-2, schools may not be a specific driver for transmission [32].

Sex, education status, employment, alcohol use and smoking cigarettes were not found to be independent factors associated with number of contacts in our study. Employment was found to influence the number of contacts in Britain, but was also dependent on type of employment [22]. A previous study within a South African community in Cape Town found differences in contact rates based on employment status and level of education, although different methods were utilized [13]. A greater number of contacts was observed for employed individuals, and those with secondary school education [13]. The high proportion of study participants which were unemployed, and low proportion with secondary education in our study may have contributed to not observing differences between these groups. This also limits the generalizability of our results to other communities.

Our study had limitations. Compared to other contact surveys preformed, we had a smaller sample size which could lead to results not being representative for the community. The advantage however is that households were randomly selected. The characterization of contact patterns in the PHIRST cohort will also be used in future research on influenza transmission patterns in these households. We only surveyed one day per participant and therefore cannot describe variability in day-to-day contacts by individual participants. We used this sampling frame to prevent fatigue and subsequent reduction in reported number of contacts as seen in other studies [33]. We were not able to measure differences in holiday and non-holiday periods. Relationships within the households were complex and capturing function within the household (breadwinner, child-caregiver, etc.) might have led to differences in contact rates, but the information was not collected for the PHIRST cohort. As a general limitation of the contact survey, we could have missed some contact events, especially in younger children and shorter contacts due to recall bias. We believe by employing the time-use survey to guide participants and interviewers through the day, missed contact events would have been reduced.

## Conclusions

We used contact diaries to describe the contact patterns in a rural and urban South African community. Age played an important role in the number and duration of contact events, with children having the highest contact rates; most of this contact time occurred at school. Children at the rural site included in our study had almost twice the contact rate than children from the urban site, and contact rates also differed based on day of the week. Our results, compared to previous studies, highlight how contact rates may differ based on the setting, and this could influence results when building transmission models. These data will be useful when building infectious disease models in similar communities to assess transmission dynamics and possible intervention strategies. A specific approach to explore would be interventions to reduce contacts, and therefore disease transmission, within schools.

## Supplementary Information

**Additional file 1.** Study population for contact survey at rural and urban site, South Africa, 2018

**Additional file 2.** Description of study population and contact rates at rural and urban site, South Africa, 2018.

**Additional file 3.** Cumulative person hours in contact reported by age, location and site, South Africa, 2018.

**Additional file 4.** Contact survey and time use survey.

**Additional file 5.** Contact survey data. (CSV 5071 kb)

## Data Availability

All data generated and analysed during this study are included in this published article and its supplementary information files (additional file 5).

## Abbreviations

95% CI: 95% confidence interval
COVID-19: Coronavirus disease 2019
IQR: Interquartile range
PHIRST study: Prospective Household cohort study of Influenza, Respiratory Syncytial virus and other respiratory pathogens community burden and Transmission dynamics in South Africa
RR: Risk ratio
RSV: Respiratory syncytial virus
SARS-CoV-2: Severe acute respiratory syndrome corona virus 2 (SARS-CoV-2)
