# Supplementary figures and images for: A cross-sectional study measuring contact patterns using diaries in an urban and a rural community in South Africa, 2018
Source: BMC Public Health. 2021 Jun 3;21:1055. doi: 10.1186/s12889-021-11136-6 (PMC8172361; doi:10.1186/s12889-021-11136-6)

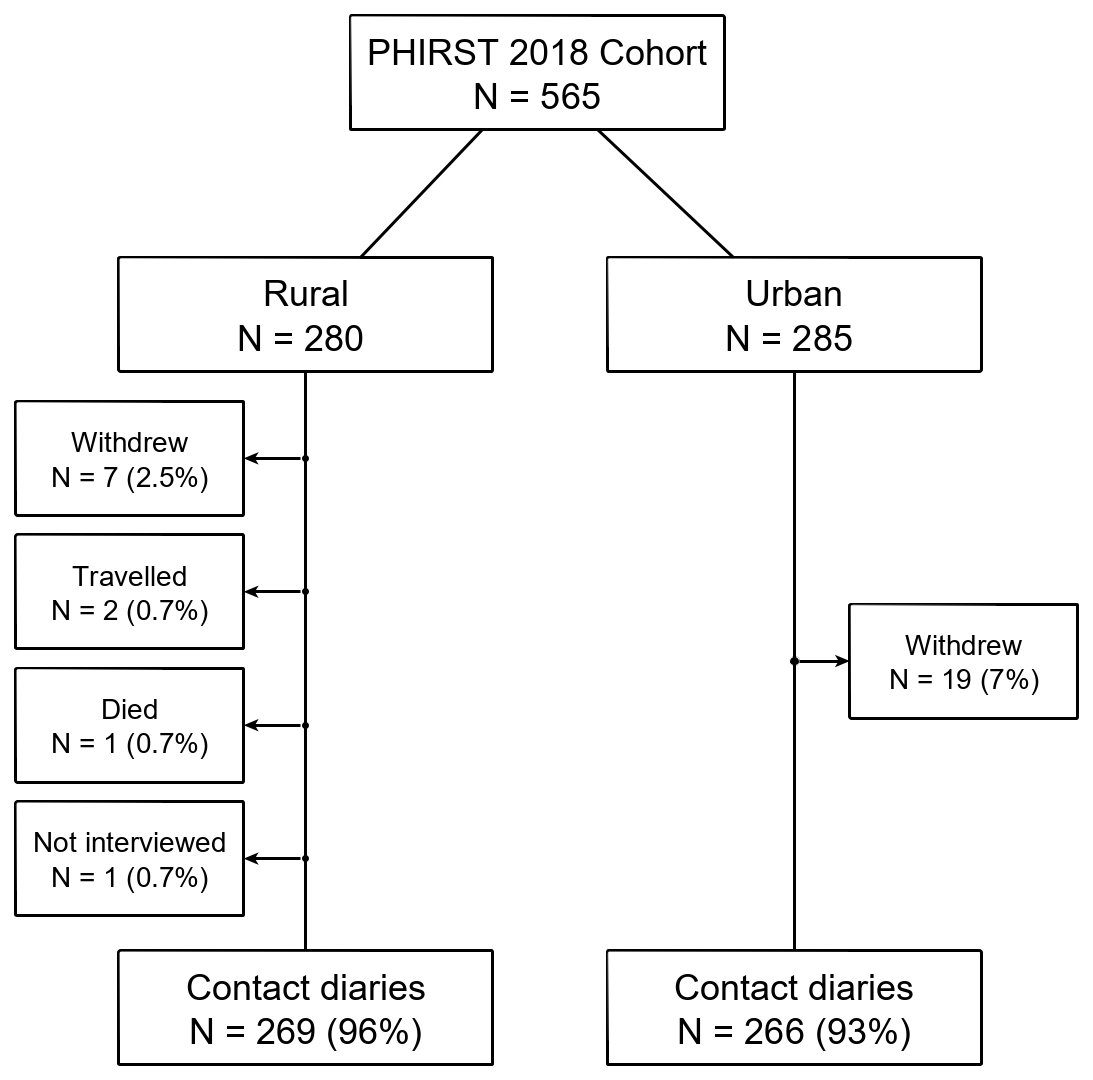

Supplement: Supplementary file 1 — Additional file 1. Study population for contact survey at rural and urban site, South Africa, 2018 [file 12889_2021_11136_MOESM1_ESM.tif]
