# Supplementary material for: A cross-sectional study measuring contact patterns using diaries in an urban and a rural community in South Africa, 2018
Source: BMC Public Health. 2021 Jun 3;21:1055. doi: 10.1186/s12889-021-11136-6 (PMC8172361; doi:10.1186/s12889-021-11136-6)
Supplement: Supplementary file 2 — Additional file 2. Description of study population and contact rates at rural and urban site, South Africa, 2018. [file 12889_2021_11136_MOESM2_ESM.pdf]

A cross-sectional study measuring contact patterns using diaries in an urban and a rural community in South Africa, 2018

Jackie Kleynhans, Stefano Tempia, Meredith L. McMorrow, Anne von Gottberg, Neil A. Martinson, Kathleen Kahn, Jocelyn Moyes, Thulisa Mkhencele, Limakatso Lebina, F. Xavier Gómez-Olivé, Floidy Wafawanaka, Azwifarwi Mathunjwa, Cheryl Cohen, the PHIRST group

Table. Description of study population and contact rates at rural and urban site, South Africa, 2018.

95%CI – 95% confidence interval

NEO – Not enough observations, contact rate only calculated for strata with 4 or more participants.

NA – Not applicable, the number of participants who reported smoking at the rural site were only 3, and therefore contact rate could not be calculated. We therefore also did not calculate the overall contact rate.

Other adult – Includes relationships of participants 18 years and older to the head of household (n): brother (7), sister (7), wife (19), husband (4), cousin (1), niece (4), nephew (2), other relative (3), not related (4).

Other child – Includes relationships of participants younger than 18 years to the head of household (n): brother (10), sister (11), wife (1), cousin (5), niece (6), nephew (9), other relative (10), not related (2).

	Overall		Rural		Urban	
	Participants (%)	Contact rate (95%CI)	Participants (%)	Contact rate (95%CI)	Participants (%)	Contact rate (95%CI)
**Overall**	535 (100)	14 (13-15)	269 (100)	21 (14-28)	266 (100)	12 (11-13)
**Age**						
< 7 years	134 (25)	15 (12-17)	86 (32)	19 (11-27)	48 (18)	9 (7-11)
7-13 years	122 (23)	21 (15-27)	60 (22)	32 (19-45)	62 (23)	17 (12-21)
14-18 years	56 (10)	22 (8-35)	32 (12)	49 (27-70)	24 (9)	15 (5-24)
19-64 years	208 (39)	12 (11-13)	85 (32)	16 (9-23)	123 (46)	11 (10-12)
≥65 years	15 (3)	8 (4-12)	6 (2)	7 (3-11)	9 (3)	9 (0-18)
**Household role**						
Head of home	112 (21)	13 (10-15)	56 (21)	13 (10-15)	56 (21)	10 (8-12)
Parent	10 (2)	9 (6-11)	1 (0)	NEO	5 (2)	9 (6-12)
Grandchild (18 years or older)	12 (2)	12 (1-22)	3 (1)	12 (1-22)	9 (3)	12 (2-22)
Child (18 years or older)	40 (7)	12 (8-16)	18 (7)	12 (8-16)	22 (8)	12 (9-15)
Other adult	51 (10)	11 (10-12)	14 (5)	11 (10-12)	37 (14)	11 (9-13)
Grandchild (younger than 18 years)	91 (17)	17 (13-21)	48 (18)	17 (13-21)	43 (16)	12 (8-16)
Child (younger than 18 years)	165 (31)	18 (14-22)	94 (35)	18 (14-22)	71 (27)	15 (12-18)
Other child (younger than 18 years)	54 (10)	16 (2-30)	35 (13)	16 (2-30)	19 (7)	11 (8-14)
**Sex**						
Male	198 (37)	15 (13-17)	95 (35)	26 (14-38)	103 (39)	13 (12-14)
Female	337 (63)	14 (13-15)	174 (65)	21 (15-27)	163 (61)	11 (10-12)
**Type of day investigated**						
Weekday	364 (68)	13 (11-15)	170 (63)	16 (13-19)	194 (73)	10 (8-12)
Weekend day	171 (32)	16 (13-18)	99 (37)	33 (23-42)	72 (27)	13 (12-14)
**Day of week investigated**						
Monday	81 (15)	14 (12-16)	24 (9)	34 (22-46)	57 (21)	12 (10-14)
Tuesday	76 (14)	14 (12-16)	32 (12)	15 (5-24)	44 (17)	13 (10-16)
Wednesday	47 (9)	29 (9-49)	29 (11)	51 (29-73)	18 (7)	19 (9-29)
Thursday	103 (19)	21 (11-31)	58 (22)	42 (32-51)	45 (17)	13 (9-17)
Friday	57 (11)	13 (7-19)	27 (10)	20 (5-35)	30 (11)	12 (7-16)
Saturday	94 (18)	13 (10-16)	56 (21)	16 (14-18)	38 (14)	11 (7-15)
Sunday	77 (14)	12 (9-15)	43 (16)	16 (7-25)	34 (13)	9 (7-11)
**Education level**						
<18 years	310 (58)	17 (14-20)	177 (66)	28 (18-38)	133 (50)	13 (11-15)
None	18 (3)	12 (6-18)	14 (5)	12 (-1-25)	4 (2)	NEO
Primary	45 (8)	13 (6-20)	20 (7)	26 (14-38)	25 (9)	9 (5-13)

Some secondary	95 (18)	11 (10-12)	22 (8)	12 (9-14)	73 (27)	11 (9-13)
Secondary completed	64 (12)	13 (8-18)	36 (13)	22 (11-32)	28 (11)	12 (10-14)
Post-secondary	3 (1)	NEO	0 (0)	NEO	3 (1)	NEO
**Employment**						
Employed	98 (18)	17 (14-20)	32 (12)	28 (18-38)	66 (25)	13 (11-15)
Unemployed	127 (24)	13 (11-15)	60 (22)	18 (7-29)	67 (25)	12 (11-13)
**<18 years**	310 (58)	11 (9-13)	177 (66)	13 (6-20)	133 (50)	9 (7-11)
Alcohol use						
No	148 (28)	14 (11-17)	92 (34)	20 (11-29)	56 (21)	13 (9-16)
Yes	116 (22)	11 (10-12)	19 (7)	11 (-1-23)	97 (36)	11 (10-12)
<15 years	271 (51)	17 (14-20)	158 (59)	27 (17-37)	113 (42)	13 (11-15)
**Smoke cigarettes**						
No	221 (41)	NA	108 (40)	NEO	113 (42)	11 (9-13)
Yes	43 (8)	NA	3 (1)	NEO	40 (15)	12 (9-14)
<15 years	271 (51)	NA	158 (59)	NEO	113 (42)	13 (11-15)
