# Supplementary material for: A cross-sectional study measuring contact patterns using diaries in an urban and a rural community in South Africa, 2018
Source: BMC Public Health. 2021 Jun 3;21:1055. doi: 10.1186/s12889-021-11136-6 (PMC8172361; doi:10.1186/s12889-021-11136-6)
Supplement: Supplementary file 3 — Additional file 3. Cumulative person hours in contact reported by age, location and site, South Africa, 2018. [file 12889_2021_11136_MOESM3_ESM.pdf]

A cross-sectional study measuring contact patterns using diaries in an urban and a rural community in South Africa, 2018

Jackie Kleynhans, Stefano Tempia, Meredith L. McMorrow, Anne von Gottberg, Neil A. Martinson, Kathleen Kahn, Jocelyn Moyes, Thulisa Mkhencele, Limakatso Lebina, F. Xavier Gómez-Olivé, Floidy Wafawanaka, Azwifarwi Mathunjwa, Cheryl Cohen, the PHIRST group

Table. Cumulative person hours in contact reported by age, location and site, South Africa, 2018.

*Total number of contacts per group / number of participants in group

	Cumulative person hours in contact / average number of contacts* (hours per contact)									
	Rural					Urban				
	<7 years	7 - 13 years	14 - 18 years	19 - 64 years	≥65 years	<7 years	7 - 13 years	14 - 18 years	19 - 64 years	≥65 years
**Home**	17.4 / 6.0 (2.9)	19.0 / 6.8 (2.8)	18.1 / 7.2 (2.5)	17.0 / 6.6 (2.6)	13.4 / 5.3 (2.5)	20.0 / 6.0 (3.3)	18.5 / 6.1 (3.0)	14.8 / 5.8 (2.5)	17.8 / 5.8 (3.0)	19.8 / 5.8 (3.4)
**School**	47.1 / 18.1 (2.6)	50.0 / 28.1 (1.8)	86.2 / 41.7 (2.1)	1.2 / 2.2 (0.6)		12.4 / 7.7 (1.6)	47.0 / 24.7 (1.9)	46.2 / 30.5 (1.5)	3.0 / 1.6 (1.9)	
**Work**				8.7 / 3.2 (2.7)				0.3 / 18.5 (0.0)	5.7 / 0.0 (698.7)	
**Transport**	0.3 / 0.7 (0.5)	0.3 / 0.4 (0.9)	1.5 / 1.4 (1.1)	1.9 / 4.3 (0.4)		1.4 / 1.0 (1.5)	0.7 / 1.9 (0.4)	0.1 / 0.4 (0.2)	1.1 / 1.7 (0.6)	7.4 / 5.7 (1.3)
**Other**	7.7 / 6.0 (1.3)	18.7 / 11.6 (1.6)	10.5 / 9.8 (1.1)	17.3 / 17.6 (1.0)	3.4 / 2.3 (1.4)	4.0 / 2.0 (2.0)	9.9 / 5.9 (1.7)	15.1 / 6.8 (2.2)	11.5 / 7.9 (1.5)	1.6 / 11.7 (0.1)
**All**	72.6 / 30.8 (2.4)	88.1 / 46.9 (1.9)	116.3 / 60.2 (1.9)	46.1 / 33.8 (1.4)	16.8 / 7.7 (2.2)	37.7 / 16.6 (2.3)	75.9 / 38.6 (2.0)	76.4 / 62.0 (1.2)	38.2 / 17.0 (2.2)	29.2 / 23.1 (1.3)
